# Quantized visual awareness

**DOI:** 10.3389/fpsyg.2013.00869

**Published:** 2013-11-22

**Authors:** W. A. Escobar

**Affiliations:** Department of Biology, Rollins Research Center, Emory UniversityAtlanta, GA, USA

**Keywords:** visual awareness, cognition, striate cortex, blindsight, agnosia, qualia

## Abstract

The proposed model holds that, at its most fundamental level, visual awareness is quantized. That is to say that visual awareness arises as individual bits of awareness through the action of neural circuits with hundreds to thousands of neurons in at least the human striate cortex. Circuits with specific topologies will reproducibly result in visual awareness that correspond to basic aspects of vision like color, motion, and depth. These quanta of awareness (qualia) are produced by the feedforward sweep that occurs through the geniculocortical pathway but are not integrated into a conscious experience until recurrent processing from centers like V4 or V5 select the appropriate qualia being produced in V1 to create a percept. The model proposed here has the potential to shift the focus of the search for visual awareness to the level of microcircuits and these likely exist across the kingdom Animalia. Thus establishing qualia as the fundamental nature of visual awareness will not only provide a deeper understanding of awareness, but also allow for a more quantitative understanding of the evolution of visual awareness throughout the animal kingdom.

## INTRODUCTION

Neuroscience has made great strides in understanding the structure and workings of vertebrate brains. Nowhere is this more evident than in describing the functional architecture of the mammalian, and more specifically, the primate visual sensory pathway and cortices. Over the past century, neuroscience has evolved from rudimentary understandings of neurons to investigating the nature of visual awareness and the neural correlates of consciousness (NCC). The NCC are defined as the minimal neural activities (circumscribed by the neural circuits and centers involved) required to generate a conscious experience (Koch and Braun, 1996; Tononi and Koch, 2008). Although great advances have been made in identifying the circuits and centers that process specific aspects of vision, it is still not clear how the activity of these circuits and centers generates our inner visual experience.

Much research indicates that a high-level of integration is required to generate our subjective experience of vision with many of the identified centers prodigiously interacting with each other (Edelman et al., 2011). This level of integration corresponds well with our daily experience of the outer world since we humans have a holistic experience of the outer world. That is to say that our inner subjective experience is not fragmented but completely integrated with depth, color, and motion all imbedded within our overall visual experience. Indeed, this holistic-level experience is so common that it is hard to conceptualize anything different. I believe our intimate connection with our personal visual experience has biased our approach in thinking about vision and the questions that are currently being asked about visual awareness in neuroscience. For example, there is an assumption that visual awareness only exists at the level of our complete human experience, but few current researchers have asked the question of whether awareness is quantized or can exist at a smaller level independent of our overall visual experience.

Many in the past have discussed the idea of quantized visual awareness (perceptual atoms) and it is clear that philosophers as far back as Hume and Descartes were considering concepts related to the questions proposed above (Julesz and Schumer, 1981; Garrett, 1997). The reason these philosophical assertions have persisted is that the concept of quantized awareness is in line with what we know about nature. Indeed, if we take the wider perspective of scientific discoveries over the past few centuries, it is clear that nature operates on this principle of quanta, and this applies to more abstract forms of nature like energy (Bohr, 1913; Planck, 1914).

All forms of energy and matter in the universe appear to be quantized. In other words, there is some smallest unit that still retains the qualities associated with whatever form one studies. Examples of this are found all around us and include: atoms (elemental matter), cells (life), and photons (energy). Given that this is a general principle of nature, it stands to reason that the same should apply to the natural phenomenon of visual awareness. Another way of stating the same is “Why should visual awareness be the exception to the rule?”

## THEORY

Although we tend to think of information in a symbolic way, in nature information is represented as structure or gradients (e.g., electrochemical). Given that the brain is a natural system, it is likely the information that leads to a visual experience has a structural component.

In the field of the philosophy of mind and in psychology, it has become routine to label inner-subjective experiences, like seeing the color blue, as qualia (Searle, 1997; Edelman et al., 2011). Thus, any personal experience of the outer world would include a great many qualia describing the various aspects of the scene in which one finds herself/himself. The hypothesis proposed here states that at its most fundamental level, visual awareness is composed of quanta of awareness or qualia^1^. Each one of these qualia is produced by neural circuits comprised of hundreds to thousands of neurons, and it is the unique topologies of these circuits that result in distinct, specific, and reproducible qualia^2^. Thus, a neural circuit with a specific topology will reliably reproduce the same bit of awareness that corresponds to perceiving the color blue, while another topology results in perceiving the color red.

The physical manifestation of a quale is not the neural circuit itself but the electromagnetic field (EMF) produced by the active neural circuits. Each unique neural circuit should produce a distinct EMF pattern and it is the EMF pattern that is the physical aspect of a quale. A corollary to this hypothesis is that the production of qualia is not dependent on the material making the circuit itself and therefore it should be possible to make synthetic qualia in the laboratory by designing circuits that mimic the topology of circuits identified in primate brains that produce qualia. This corollary marks a significant break with philosophical proposals made in the past about perceptual atoms or qualia (Julesz and Schumer, 1981; Llinás and Churchland, 1996)^3^.

The contribution to our overall visual experience of each quale is small. A metaphor used in a previous communication is that we can consider each quale as a pixel in a computer screen (Escobar, 2011). The big difference being that a quale does not represent a specific color but awareness of the color itself. Moreover, qualia are so small compared to our overall visual field that they are hard to experience individually. Our subjective visual experience requires the integration of a large number of these qualia, and this integrated state is the level of experience that correlates most closely with our common everyday vision.

Although it may appear that this hypothesis requires a multiplicity of different types of qualia, it is likely a few diverse types could be used to create a great number of different visual states. For example, we might only need three color qualia to synthesize all perceived colors in the same way red, blue, and green pixels are used to create a variety of colors on computer screens. Besides color qualia, there must also be qualia types that correspond to different aspects of vision. For instance, there are likely qualia that produce the sensations of depth, motion, or orientation. Once again, varying the number and combination of these few qualia types could create a large variety of experiences.

The ideas described in the previous paragraph parallel the structure and function of biological systems. A countless variety of proteins in biological systems with wide-ranging properties result from covalently linking varying numbers of the same twenty amino acids. The complex and highly structured tissues and organs of our bodies are created from a limited set of approximately 200 cell types. Recombining four distinct nucleotides into diverse nucleic acid sequences creates the immense number of genes found in biological systems.

The reason this approach is employed at all levels in biological systems is that it allows for the production of a vast number of complex structures from a limited set of building blocks. This maximizes structural diversity with a minimal investment of energy. Given the constantly changing, highly diverse environments we encounter, it seems likely that the same evolutionary pressures that have produced the complex structures of our bodies would also select for a visual system that could represent a vast number of possible environments from a limited set of qualia building blocks.

Not all neural circuits produce qualia. I believe that most do not with clear examples of these being the circuits that produce automated responses like the knee-jerk response. Qualia require circuits with specific topologies containing hundreds to thousands of neurons and even all of these do not result in qualia. We are born with a surplus of neurons and circuits in our brains. Circuits that are activated through our experience are retained while those that are not are diminished. Gerald Edelman has described this process as neuronal group selection (Edelman, 1992; Edelman et al., 2011). This concept must apply to qualia and we can say that neural circuits producing qualia that effectively describe our environment and aid in our survival will be enhanced and maintained while those circuits that do not will be degraded.

Although I do discuss ideas about how qualia are initially integrated in order to understand how this qualia proposal fits in with what is known about the circuitry of primate visual cortices, the process of binding various aspects of vision into an overall visual experience is not the focus of this paper. There are various schools of thought on how different attributes of what we perceive are bound together; for example, the ideas of Crick and Koch on temporal synchronization (Crick, 1995; Llinás and Churchland, 1996; Crick and Koch, 2003; Edelman et al., 2011). This issue of binding, however, is altogether a different question than whether or not visual awareness is quantized or whether individual quanta of awareness can serve as fundamental units of visual awareness.

The model proposed here has the potential to shift the focus of the search for visual awareness to the level of microcircuits. Obviously, this would have implications for the fields of neurobiology, psychology, and philosophy of mind, but beyond these the idea of quantized awareness could open the doors to a quantitative discussion of the evolution of awareness. The focus is no longer the maximally complex central nervous systems of mammals or primates but perhaps any organism that displays these microcircuits (no matter how simple the organism). In other words, the discussion of awareness becomes much more expansive since we can look for and identify certain neural circuit topologies throughout the animal kingdom, and in the process, create a phylogeny of visual awareness.

## EXPERIMENTAL SUPPORT

Support for the existence of qualia is given by the detailed structure of the cerebral cortex itself. When probed at microscopic scales, we find the cortex is composed of a myriad of local circuits that are communicating with each other. Although many centers have been identified in the visual cortex, each of these is composed of many microcircuits that process information locally and then send off their data to other centers or communicate with other local circuits. Ramón y Cajal (1899) and other researchers observed this architecture over a century ago.

More recently, the work of David Hubel and Torten Wiesel demonstrated the presence of a large number of highly organized neural circuits they referred to as ocular dominance columns (ODCs; Hubel, 1959; Hubel and Wiesel, 1959, 1968). These columns are packed tightly together in the part of the visual cortex known as the primary visual cortex (V1 or striate cortex), and each corresponds to a specific location in our visual field (Hubel et al., 1978; Cheng et al., 2001; Adams et al., 2007). V1 is the first cortical structure to receive visual information, and it is believed to operate at a rudimentary level in generating vision. Despite its basic role, however, V1 is thought to process the initial stages of various aspects of vision like color and motion. For example, the complex cells found in ODCs respond most actively when a line of a specific orientation moves through their corresponding receptive field. Moreover, Hubel and Wiesel identified areas of V1 known as blobs that respond strongly to colored stimuli (Hubel, 1983).

The qualia model proposed here offers that these well-known and described ODCs are more than way stations for visual information and serve as the seat of visual awareness. That is to say that activated ODCs found in V1 of several primates (including humans) produce bits of awareness corresponding to color, motion, orientation, or depth and that the highly organized structure of V1 itself allows for these qualia to be appropriately mapped into our overall visual awareness (Adams et al., 2007). Most importantly, these bits of awareness can exist independently of whether they are integrated into a conscious visual experience.

This proposal forces us to think about awareness in a fundamentally different way because we must consider that it is possible to have bits of awareness within our striate cortex that are independent of visual perception. There seems to be a contradiction here since how can there be any visual awareness that is independent from our conscious experience? Most current models that aim to explain visual awareness do not allow for this condition. However, there is a form of visual awareness that is not well understood and seems to demonstrate these properties in humans. This odd case of perception was first described in the 1970s by Weiskrantz and others and is known as blindsight (Weiskrantz et al., 1974; Koch and Braun, 1996; Milner, 1998; Tong, 2003).

Blindsight occurs when lesions to V1 completely eliminate conscious visual perception. Individuals with Type 1 blindsight report they can no longer perceive visual stimuli including seeing motion, color, shapes, or any other visual cues. A typical experiment would be to ask a patient to choose the direction of a right or left moving object. Strangely enough, when blindsight patients are probed under forced-choice conditions, they respond with the correct answer at a frequency greater than chance regarding visual stimuli (Azzopardi and Cowey, 1998). Indeed, these patients are often surprised by their success rate in these forced-choice experiments. Thus, it appears that individuals do retain a form of awareness that is not directly tied to the patient’s experienced perception. Although blindsight is most often associated with activity in extrastriate cortices, it nonetheless demonstrates that a form of awareness can exist independent of conscious experience.

Lamme (2001, 2003) has proposed a model for the production of conscious visual experiences. In his model, he separates the feed-forward sweep (FFS) of the geniculocortical pathway from the recurrent processing that follows the activation of higher visual centers. The FFS is the rapid (~50 ms) progression of neural signals through V1 and onto higher centers like V3, V4, V5, and the inferior temporal cortex (ITC). Recurrent processing occurs only after a region is activated by the FFS (100–150 ms). According to Lamme, the FFS is an unconscious process while recurrent processing (for example, from V4 or V5 to previously activated centers like V1) results in phenomenal consciousness. Phenomenal consciousness relates to an experience that is not fully conscious. An example is given by Block when he describes the sensation one has when the motor of a refrigerator shuts off and one has the impression that it has been on for a while previous to that moment (Block, 1996). In other words, the experience was there but not fully accessible to consciousness (access consciousness).

I agree with Lamme’s assertion that recurrent processing results in phenomenal awareness and I would even agree with Lamme that the FFS is unconscious, as we tend to think of consciousness. The model I am proposing here, however, states that awareness is produced by the FFS but it is in the form of individual bits of visual awareness – unintegrated and not experienced as a whole. The model holds that these individual bits of visual awareness (qualia) exist independently of whether they are integrated into a phenomenal conscious experience. Moreover, it is the integration of a subset of all of these independent qualia through recurrent processing that results in phenomenal consciousness. This proposal requires us to think about consciousness in manner that is bottom-up instead of top to bottom. Here we start with bits of awareness (qualia) uniting to create a larger more comprehensive form of awareness that we can think of as a high-level primate visual experience.

Cases that support this perspective come from studies of patients with visual agnosia. These patients have lost the ability to recognize familiar objects and, in some cases, lose the ability to recognize even simple shapes. Milner reports the case of patient D.F. who suffered from a severe form of agnosia resulting from carbon monoxide poisoning. D.F. retained an intact V1 and demonstrated an outstanding level of visual acuity as evidenced by her ability to distinguish between gray patches and a fine pattern of dots (Milner, 1991). Also, Zeki reports the case of agnosia in a stroke victim resulting in a severe lesion of the prestriate cortex (V2) while retaining an intact V1 (Zeki, 1992). This individual could draw local features of objects (corners, line segments of specific orientation) but did not retain the capacity to understand what was drawn. This patient could even draw structures as complex as St. Paul’s Cathedral in London and yet not understand the figure he had just rendered. Both of these examples demonstrate that an intact V1 yields awareness of local, small features as would be expected if activated ODCs were creating qualia associated with specific locations of the visual field. Visual perception did arise in these patients, but this perception seemed to manifest itself as independent bits of visual awareness corresponding to local features. Since V1 remained intact in these patients, we can infer that the integration of these bits of awareness must occur through the action of extrastriate cortices.

It is well established that extrastriate centers of the visual cortex play a role in processing high-level visual information: V5 is associated with motion processing, V4 plays a role in color and form processing, and V3 activity relates most closely to dynamic form processing (Zeki, 1978). In the model proposed here, these centers are synthesizing these high-level experiences by recruiting and integrating individual qualia produced in V1. A simple way of thinking of this is that V1 provides the palette that is used by the extrastriate centers (V5, V4, or V3) to paint our perceptive canvas. In addition, when these same centers emphasize or diminish the contribution of certain V1 qualia, it is possible to focus our perception on different aspects of the scene we see.

**FIGURE 1:**
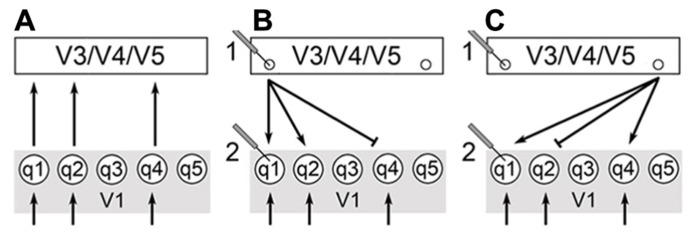
**V1 receives stimuli from the geniculocortical pathway as indicated by the arrows below.** V3/V4/V5 corresponds to a generalized higher visual center and q1-5 are qualia produced in V1. **(A)** Qualia from V1 supply a surfeit of information in feedforward pathways (FFS) to these higher centers. **(B)** Extrastriate calculations select for output cells indicated by small circles. The choice of output cells is key for the selection and integration of specific qualia from V1. **(C)** Changing recruitment patterns of extrastriate centers allow for changes in perception – compare to **(B)**. This can be monitored physiologically as shown by electrodes 1 and 2. The output of calculations in extrastriate centers manifests as output cells with specific recurrent pathways back to V1. Thus, we would expect the activity of cells in extrastriate centers to change more significantly than V1 since the choice of output cells changes as calculation outputs and percepts vary [compare electrodes 1 and 2 in **(B,C)**]. In addition, V1 cells continue to receive input from the geniculocortical pathway and this would moderate changes in activity of V1 cells.

There is a significant level of recurrent communication that occurs between V1 and areas like V3, V4, and V5. It is likely that the back projections from these extrastriate regions play a role in integrating the qualia produced in V1. V1 activity is modulated by extrastriate centers as monitored by functional magnetic resonance imaging (fMRI; Kosslyn et al., 2001; Tong, 2003). Mehta (2000) has shown that attentional modulation of neural activity occurs after the initial transient response of V1, and that recurrent activation of V1 occurs after attentional effects of V4 and the ITC. An interpretation is that feed-forward pathways from V1 to extrastriate cortical regions supply a surfeit of information and that recurrent pathways play a role in selecting and integrating the bits of awareness used for conscious perception (**Figure 1**).

This ties in well with the work of Logothetis and others demonstrating that cells of the macaque monkey’s V1 do not respond well to changes in perception due to binocular rivalry (Leopold and Logothetis, 1996; Sheinberg and Logothetis, 1997). These studies present different and competing images to either eye of primates that have been trained to respond when they see one image or another. The primate pulls a lever or otherwise indicates when it sees one image over the other while electrodes indicate the level of activity of specified cortical cells. In this way, researchers can tell when the primate’s perception has shifted and can correlate this shift to changes in the activity of individual neurons. Logothetis and others have shown that ~90% of ITC cells, ~40% of the cells of V5, ~40% of V4 cells, and <20% of V1/V2 cells correlate to changes in perception. Many have interpreted these results to indicate that the macaque V1 is not playing a direct role in perception since it appears that so few cells respond to changes in perception.

In macaques, integration of individual qualia may depend on changing extrastriate recruitment patterns and not in modulating the activity of individual ODCs within V1 (Kosslyn et al., 2001). Thus, ODC activity should not change in response to binocular rivalry since ODCs only become important to high-level visual experiences as individual qualia are recruited by extrastriate centers that integrate these bits of awareness and create our overall perception. Referring to the painting example above, imagine that a painter is working on two canvases at the same time. The paints on the palette do not appear or disappear in a canvas-dependent manner. The paints remain on the palette even as the painter goes back and forth between canvases. The palette represents V1 activity. The extrastriate centers correspond to what is happening on the canvases. If you monitor activity at one of these canvases, you will notice that the activity comes and goes depending on whether the painter is working on that canvas. However, the activity of the palette remains constant and does not change with the choice of canvas (**Figure 1**).

Binocular rivalry studies in humans using fMRI have shown a modulation of V1 in response to changes in perception. In humans, recurrent pathways may play a greater role in modulating the activity of ODCs, and perhaps a mix of changing recruitment patterns of extrastriate cortices and modulating ODC activity is used to accentuate the contributions of specific ODCs in a given percept (Polonsky et al., 2000; Tong and Engel, 2001).

## DISCUSSION AND COMPARISON

In the “neurobiological theory of consciousness,” Francis Crick and Christof Koch propose the ~40 Hz oscillations observed in the cerebral cortex are the means by which disparate bits of information are bound together and that this oscillatory process contributes to the formation of a conscious experience (Crick and Koch, 2003).

Crick and Koch’s theory of consciousness does not allow for awareness to exist at smaller levels (i.e., qualia) and considers all the information-processing preceding binding to lack an explicit experiential component. The qualia model differs with this oscillatory-binding theory in that qualia are considered independent units of awareness that exist whether or not they are incorporated into a conscious experience. However, I believe it is difficult for humans to experience an individual quale and that our visual phenomenal consciousness only arises after a significant number of these qualia have been integrated or bound together. It is possible that the mechanism for the global-level binding of qualia is the oscillatory mechanism proposed by Crick and Koch, and consequently, these two theories may dovetail together at this level (see below).

Many have argued that the neural activity of V1 cannot play a significant role in the direct experience of visual consciousness (Silvanto, 2008; Tononi and Koch, 2008). For example, the modulation of neural activity in higher visual centers like V5 or V4 in response to changes in perception seem to indicate an active role in seeing a stimulus while the lack of perceptual modulation in V1 activity in macaques indicates the striate cortex has little to no role in the visual experience (Leopold and Logothetis, 1996). As I have previously proposed, this result could be interpreted as changing recruitment patterns of extrastriate regions with V1 providing the basic units (qualia) for recruitment.

A strong argument made by Zeki and Bartels (1999) against the necessity of V1 in direct conscious experience is the well-documented cases of visual awareness arising in patients with blindsight. Since blindsight patients have significant lesions in V1, this special visual awareness (e.g., sensing fast motion) is thought to bypass V1 through subcortical pathways. I would contend that these studies indicate that some qualia may exist outside of V1 but that this is the exception to the rule and not the general case. For example, it is easy to imagine that a rapid pathway for generating the sensation of fast motion from external stimuli would yield a significant selective advantage to organisms that possessed it. The split second saved in such a case would elicit the fight or flight response all the sooner and could make a difference in surviving a predatory attack. It is possible there are other exceptions to the rule of qualia in V1 that yield other advantages but these are special in nature and not the general case.

Unlike many of the studies that try to associate visual experience with extrastriate visual centers and beyond, Lamme has taken a different approach and argued that it is not the specific location as much as the direction of processing that matters. In his model, the rapid flow of information in the feedforward sweep (FFS) of the geniculocortical pathway is an unconscious process while the recurrent activation that occurs after an area is activated by the FFS creates at least phenomenal consciousness. This is in line with Mehta’s work, which demonstrates that attentional modulation of neural activity occurs post the V1 FFS and is more closely related to the recurrent activation of V1 by V4 and ITC (Mehta, 2000).

In addition, Lamme’s model proposes that phenomenal awareness is gated by attention and that attention allows phenomenal consciousness to move into access consciousness (Lamme, 2003). As its name implies, this form of consciousness provides access (verbal and other motor control) to the items contained within it and it is what is most commonly understood as a conscious experience. Lamme’s model supports the idea that there are different levels of consciousness.

The model I am proposing here holds that there is at least a third level of “conscious” experience or awareness. This form of awareness is very simple and small compared to our overall visual experience but it exists as awareness whether it is integrated into a larger experience or not. These bits of awareness are found in large numbers in V1, although this does not preclude them from existing in extrastriate cortical regions. Each quale is so small in comparison to our usual visual experience that it may not seem to be a form of awareness and this coincides with Lamme’s interpretation of the FFS being unconscious.

Thus the FFS through V1 results in the production of qualia associated with specific aspects of the scene we see – color, depth, motion, and orientation at certain locations in our visual field. These are for the most part independent of each other and remain so until extrastriate centers, through recurrent processing, recruit, and integrate them into phenomenal sensory experiences. This is an important point to make again. Individual qualia do not correspond to phenomenal consciousness. It is only after many individual qualia are integrated that phenomenal consciousness arises. Changes in recruitment patterns of V1 qualia by extrastriate centers result in different percepts being produced (e.g., binocular rivalry) and it is these percepts that are competing for entrance into access consciousness.

In their theoretical paper, Zeki and Bartels (1999) argue for the existence of microconsciousnesses. This proposal parallels the idea of qualia put forth in this paper in that we are all postulating the existence of smaller forms of visual awareness that come together and contribute to the more complete form of vision we experience. Zeki and Bartels base their proposal on various features of visual processing including the observation that several perceptual aspects like color, grating orientation, and motion arise at different times. These authors have shown that the asynchronous perception of these visual attributes (30–40 ms between each) cause test-subjects to improperly associate visual cues that correspond to these visual attributes. They use these results along with a number of human studies looking at achromotopsia and akinotopsia to state that these attributes are being produced and perceived independently of each other. Therefore, each perceived visual attribute corresponds to an independent microconsciousness.

Zeki and Bartels believe these microconsciousnesses arise from the processing that takes place within each system devoted to processing these specific attributes. For instance, the system devoted to processing color, beginning with the blobs of V1 and continuing specifically through the thin stripes of V2 and V4. Processing within this and other visual systems is known to be hierarchical in that the cells activated by the FFS implicitly contain the information of cells feeding into that cell. One consequence of this multistage integration is that cells further along in the system (compare V4 to V1) have much larger receptive fields.

Zeki and Bartels propose that the nodes of the color system (e.g., the thin stripes of V2 and V4) produce microconsciousnesses that incorporate what came before in the FFS through a binding process they call generative binding. Thus, processing in higher centers like V4 produces a microconsciousness that explicitly contains previously produced microconsciousnesses due to the architecture of the color system. This would also apply to the other systems devoted to attributes of vision like motion.

In contrast, the qualia model proposes that qualia (bits of awareness) are produced in V1 by the FFS but not in the higher visual centers. Instead, these higher centers use the implicit information contained by them to inform the calculations taking place in these centers with the eventual goal of selecting specific reentrant pathways back to V1. Indeed, this is likely the reentrant activation described by Lamme that results in phenomenal consciousness.

Another important difference between the two hypotheses is that Zeki and Bartels never specifically define the neural structures associated with producing microconsciousnesses. A core aspect of the qualia hypothesis (as in nature) is that information always corresponds to a definite structure or gradients of some form. Qualia are described here as the bits of awareness produced by neural circuits with specific topologies. These circuits exist in abundance in the striate cortex and are known as ODCs. By defining the basic units of awareness in this way we create a condition that allows for experimentation and a testing of this hypothesis (see the section on proposed experiments).

**FIGURE 2:**
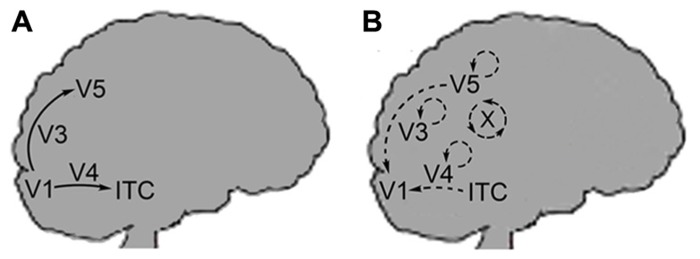
**(A)** Arrows indicate the feedforward sweep (FFS) moving from the V1 onto extrastriate centers. FFS connectivity is of the “like with like” form. **(B)** Back arrows indicate recurrent activation of V1 from extrastriate centers: V3, V4, V5, and ITC. Circular arrows indicate lateral connectivity within each center. X corresponds to the cross-talk (lateral circuitry) between the centers. Recurrent and lateral connectivity is of the “diffuse” form as indicated by Zeki and Bartels.

Furthermore, Zeki and Bartels differentiate between the neural pathways of the FFS and the lateral or reentrant circuitry back to V1 (**Figure 2**). Lateral circuitry being the interconnections between the various centers (e.g., V3, V4, V5) or different areas of the same center. The authors state that the FFS connects cells that process similar types of information they term “like with like” activation. Thus, cells of V1 tuned to movement of a specific direction will activate cells further on in the FFS that are tuned to movement in the same direction. This “like with like” activation clarifies why the FFS does not integrate the disparate qualia produced in V1 but leaves qualia independent at this stage of processing. In contrast, lateral or reentrant circuitry is more diffuse in its activation and results in the physical integration of unlike stimuli (Shipp and Zeki, 1989 – both). The authors call this integrative binding and they propose that this is the means by which microconsciousnesses are brought together.

The reentrant activation of V1 allows for the low-level integration of qualia. However, the function of lateral pathways within and between higher visual centers is to calculate which cells of V1 are activated by the higher centers and not for binding microconsciousnesses. Color constancy is a case in point. In this case, the “like with like” activation proceeding through the FFS would need to be modified since the perceived color for a given location of the visual field is different than that indicated by the light entering the eye. The “cross-talk” occurring in the higher centers through lateral pathways allows for these calculations to be made and activates the appropriate reentrant pathways to V1 from V4.

**FIGURE 3:**
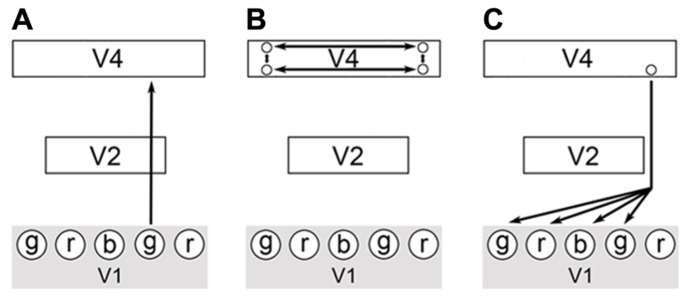
**A possible circuit describing color constancy.** **(A)** A colored stimulus elicits the FFS through the blob cells of V1, thin stripe cells of V2 and onto the thin stripes of V4. **(B)** Complex calculations of illumination ratios select for the activation of a specific output cell or set of cells from V4. **(C)** Each output cell will have a specific feedback pathway back to V1. Activation of this feedback pathway will modify the activity in V1 in specific ways and allow for the local integration of proximal qualia as part of the same feedback process. The modulation of V1 circuit activity results in qualia achieving the correct oscillatory range or to fall out of the correct range for binding to occur.

To demonstrate these points, I will describe a hypothetical visual stimulus in which a green colored object is darkened by a shadow. Imagine that combining just three types of qualia produces all perceived color: red, blue, and green. In the process of visualizing a scene, green wavelength light impinges at a certain location of the visual field and a green quale is produced at the corresponding location of the striate cortex. This results from the activation of the respective areas of V1 (ODCs in blobs) by the visual processing pathway leading from the retinas to the occipital cortex. Next, the color information is passed through the FFS up through the thin stripes of V2 and onto V4. In V4, lateral processing indicates that this location is in shadow through calculations of the illumination ratios of the scene (**Figure 3**). The output of these calculations is the activation of a specific cell or set of cells in V4 with the appropriate reentrant neural pathways leading back to V1. These pathways accentuate the original green qualia in V1 and also modulate the activity of other local circuits that produce green, red, and blue qualia. The extra qualia combine to form the equivalent of white light and in combination with the original green quale, produce a lighter shade of green^4^. The proximity of these qualia within V1 (all corresponding to the same attribute – i.e., color) and the modulation of their activity, allow for these qualia to be bound at a low-level. As stated above, reentrant pathways are diffuse and this allows unlike qualia to be physically integrated as part of the same activation process. Due to the well-known structure of V1, the spatial relationship of all qualia is implicitly encoded within each quale produced and proximal qualia can be integrated at a local level since they represent close points in the visual field. Note that this spatial synchronization of qualia still allows for the temporal asynchrony observed by Zeki and Bartels since different attributes of vision (motion, grating orientation) can arrive independently at V1.

Keliris et al. (2010) have recently published a paper studying perceptually correlated modulation of V1 activity. By using binocular flash suppression they were able to monitor the activity of neurons in macaques and correlate these modulations of neural activity to changes in perception. Monkeys in these studies were shown gratings with orthogonal orientations to either eye at the same locations of the visual field. Similar to previous binocular rivalry studies with macaques, the authors found that approximately 20% of V1 cells demonstrated changes in their spiking frequency as a function of perceptual changes.

This level of modulation is what we would expect if we were trying to modify a percept and not necessarily create a new one as described above in the example for color constancy. Reentrant pathways modulate the activity of V1 and either enhance or suppress the spiking frequency of cells. It is possible that these modulatory effects push circuits into or out of the correct oscillatory range (Crick and Koch – see above) for binding to occur. As circuits enter a suitable oscillatory range, their respective qualia are bound to the percept. Conversely, when circuits fall out of the appropriate oscillatory range, their corresponding qualia are removed from the percept. Thus, modulation of activity in V1 leads to the integration and binding of the specific qualia to be included in a percept. At the point that a threshold number of qualia are bound, phenomenal consciousness arises. From this description we can see that phenomenal consciousness is quantitatively and qualitatively distinct from the individual qualia contained within it. Beyond the large number of qualia that comprise phenomenal consciousness, this form of consciousness also includes an implicit awareness of the spatial and temporal relationships of the qualia that contribute to it.

In the 1980s Bernard Baars proposed that consciousness arises through the action of the global workspace. He originally presented this idea in his book *A Cognitive Theory of Consciousness* (Baars, 1988). As he described it, the global workspace serves as a centralized location from which disparate information processors can retrieve information and to which they can broadcast their output. Although the information processors are not conscious in and of themselves, the global workspace is conscious and it contains all the diverse elements of consciousness. Baars’ cognitive psychological approach to describing consciousness has been extended by others (Dehaene and Naccache, 2001; Dehaene et al., 2006) to include models that reproduce some proposed properties of a global workspace. For example, Dehaene et al. (2006) specify that activation of associative areas like the prefrontal and anterior cingulate cortices create a reverberating neuronal assembly with a long lasting reverberation that extends temporally past the initial stimulus. In addition, the authors define states (subliminal, preconscious) that are precursors to access consciousness (global workspace) but whose content does not necessarily enter access consciousness.

Although the global workspace model addresses many of the resonant, associative, and other properties thought to be part of access consciousness; it does not address what it is about these processes that result in conscious awareness. Consciousness is attributed to the global workspace and when unconscious information processors supply their contents to the global workspace (access consciousness) this information becomes conscious. But what makes the global workspace conscious?

The qualia model avoids this problem because it proposes that the information reaching access consciousness is already aware or conscious (qualia, phenomenal consciousness). The question ceases to be “How does information become conscious as it moves from non-conscious preprocessing centers to access consciousness (the global workspace)?” and becomes “How are smaller, independent forms of consciousness (qualia, phenomenal consciousness) incorporated into access consciousness?”

Most importantly, the quality that is changing here is whether the material contained by a conscious state is reportable. Dehaene and others maintain the belief in the existence of consciousness without the ability to report is based on the illusory “intuition that visual awareness includes a richness of content that goes beyond what we can report” (Dehaene et al., 2006). However, it is clear from cases of locked-in syndrome that conscious states can exist without report. Individuals with total locked-in syndrome have lost all voluntary muscle control but remain conscious (Schnakers et al., 2009). Thus conscious states can exist without the ability to report and reportability is not a requirement for consciousness.

**FIGURE 4:**
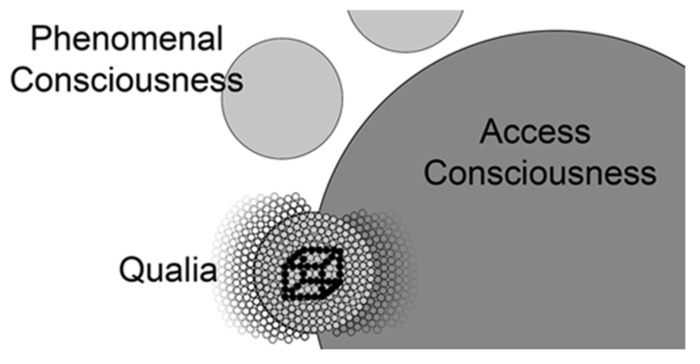
**A conceptual map of the relationship of qualia, phenomenal consciousness, and access consciousness.** Individual qualia (small circles) produced in V1 are bound together into phenomenal consciousness (larger light gray circles) by achieving the correct oscillatory range. Note the phenomenal conscious state shown below in this figure excludes many qualia that are not incorporated into this state (small circles to the left). Lamme and others postulate entrance into access consciousness (largest dark gray circle) is gated through attention and it is likely these phenomenal states (other light gray circles) compete for entrance into access consciousness (e.g., Necker cube). As phenomenal consciousness becomes part of access consciousness, it joins with the many qualia (small circles to the right) that are already part of access consciousness. Models describing the global workspace (Dehaene et al., 2006) include properties (resonance between prefrontal areas and posterior visual centers) that address the mechanism by which visual phenomenal conscious states are integrated into access consciousness.

In biological systems, we find basic units coming together to form more complex systems, which in turn contribute to systems of even greater complexity. These systems all contain top-down and bottom-up control mechanisms and at the highest levels result in very sophisticated processes that seem quite different from the properties of their basic units. All organs of the human body adhere to these principles and evolution has selected for all of these systems to operate in this way. The qualia model proposes that awareness operates in a similar fashion. Here we have the basic units (qualia) coming together to form the more complex phenomenal consciousness, which itself contributes to access consciousness. Access consciousness has the unique property of reportability due to its access to motor centers but this does not negate the existence of independent and simpler forms of visual consciousness or visual awareness in previous steps along this pathway (**Figure 4**).

## PROPOSED EXPERIMENTS

If qualia exist, their corresponding circuits must be observable in biological systems. I believe that qualia circuits or parts of qualia circuits have already been detected, but the appropriate theoretical framework has not existed to completely interpret these results. I have argued that qualia exist in the striate cortex, but they may exist in other areas throughout the brain. The microscopic scale that corresponds to the activity of a few hundred to thousands of cells is bordered by technologies used to study the activity of a few cells (electrodes) and larger regions of neural activity: positron emission tomography (PET), fMRI, and magnetoencephalography (MEG). The rate of advancement in the field of detecting neural activity is such that it will become possible in the near future to detect the activation and topology of individual circuits.

The first step in this line of research would be to determine if there is indeed a recurring set of ocular dominance column topologies within the human striate cortex. Given recent advances in imaging technology, it should be possible to do this within the next decade if not sooner. For example, the Human Brain Project has produced a detailed map of the human brain by generating 7400 slices (20 μm thick) of a human brain and then imaging each through histological staining and microscopy. Slices were digitized and reassembled *in silico* to generate a cellular level map of the brain (Amunts et al., 2013). This massive database is publicly available and might prove suitable for the fine-grained structural analysis required to identify repeating circuit topologies. Another example of a publicly available database is The Human Connectome Project (HCP). HCP provides diffusion tensor data (DTI) of the axonal connections between centers of the cerebral cortex (Rosen et al., 2010). This project may prove useful in probing the connections between the higher visual centers and V1 in great detail. One desired product from these fine-grained studies is a list of the number and type of unique circuit topologies represented in the striate cortex.

Given the existence of a recurring set of neural circuit topologies within the striate cortex, the next step would be to establish a correlation between specific visual stimuli and each of these recurring circuit topologies. An example would be to shine points of light of a certain color (red, blue, or green) at specified locations of a subject’s visual field. It should be possible to determine the structure of activated circuits within the striate cortex by using techniques like high-field fMRI. Key to this line of research is the reporting of the observed color that would allow identified neural circuit topologies to be correlated to internal subjective experiences. Of special interest in these studies would be to see if combining the activity of two distinct neural circuits can produce colors. For example, can activating circuits previously associated with the colors red and green create the subjective experience of the color yellow?

Although high-field fMRI would allow researchers to correlate specific circuit topologies to reproducible internal experiences (qualia), determining causation would require the use of transcranial magnetic stimulation (TMS) to precisely disrupt certain circuits implicated in producing qualia. This would allow researchers to see if eliminating the activation of certain circuits interrupts the production of the visual experience. Conversely, it would be interesting to investigate if the specific activation of ODC neural circuits (with TMS) of known circuit topologies would result in the subjective experience of exclusively one type of colored light.

To demonstrate that these circuit topologies are found throughout the animal kingdom, these studies must be extended into model systems like zebra fish. Muto et al. (2013) have recently demonstrated that it is possible to visualize circuit activation through transgenic expression of GCaMP. This research group observed Ca^2^^+^ transients produced in embryo zebrafish in the absence and presence of their natural prey paramecium. As a result, these researchers identified the specific circuit structure activated by the paramecium. In a similar fashion, it should be possible to determine if circuits with specific topologies are reproducibly activated in response to colored stimuli and to catalog the structures of these circuits. Studies in other model systems like mice are also producing descriptions of the detailed structure of visual circuits (Bock et al., 2011). In their recent paper, Bock et al. (2011) described the structure of activated visual cortex circuits through the combined use of light microscopy with the calcium indicator BAPTA 1-AM (OGB) followed by serial-section transmission electron microscopy (TEM). Through these techniques, Bock et al. (2011) generated a line-orientation preference map with detailed structural information about the neural cells and circuits within this section of cortex. In the same way, it should be possible to generate a color preference map that gives detailed structural information about the circuits activated by color stimuli.

The proposed qualia hypothesis predicts that at least some of the same circuit topologies activated by visual stimuli in humans should be found in various animal models since these topologies are not species specific. By creating a catalog of circuit topologies across many species and relating them to specific stimuli, we will create the foundation for understanding visual awareness and perhaps primary sensori-motor consciousness in general. It will also become possible to ask questions relating to the evolution of visual awareness and the distribution of qualia throughout the kingdom Animalia.

## CONCLUSION

Over the past few decades, there have been numerous attempts to understand visual awareness through elaborate top-down mathematical models. For example, Giulio Tononi has borrowed concepts from the field of information theory and puts forth the idea that we produce specific internal impressions by a process of making a large number of distinctions. “Not this but that” repeated many times results in only one possible answer Tononi, 2008;Tononi and Koch, 2008). It may be that these ideas accurately describe the means by which we recognize percepts, but I believe this type of computational process must occur at later and higher stages of image processing than the production of qualia. Qualia are produced early and create the fundamental percept first. As these percepts enter access consciousness they can then be compared to previously experienced percepts and this would require the types of distinctions that Tononi describes. So it may be possible these two hypotheses are complementary and not mutually exclusive.

In contrast to the several top-down approaches proposed over the past few decades, the model I put forth here is a bottom-up proposal. In some ways, this model aligns with Chalmers’ idea that there are fundamental entities in nature that need to be accepted as such (Chalmers, 1995). He postulates that consciousness is one such case and I believe this manifests as a fundamental unit of awareness defined here as a quale. In this case, it is the topology of neural excitation itself that is a simple form of awareness. Like any other biological system it is the structure that determines the function. This tenet holds throughout all biological systems and I believe this serves as a significant argument for the same applying to awareness.

Quantized visual awareness is consistent with what we know of biological systems and the generation of complexity. Basic sets of building blocks are used to create a huge variety of intricate structures in biology. Given the unpredictable and incalculable number of visual states produced in primates, it seems logical that an approach that used basic building blocks like qualia would prove advantageous.

This hypothesis enhances our ability to study visual and other forms of awareness. By establishing the existence of quantized awareness, we are liberated to look for and catalog the various forms of qualia and eventually analyze each individually to understand how their form leads to function. This approach puts awareness squarely into the realm of established scientific principles and approaches.

Qualia are likely found throughout the animal kingdom. We know there are a wide variety of nervous systems across the kingdom Animalia. However, this does not preclude the existence of rudimentary forms of awareness in many of these animals. We no longer need a whole brain but just small circuits of hundreds to thousands of neurons with specific topologies. Indeed, this may be at the heart of the evolution of visual awareness. Paleobiological studies clearly show that early animals were small and much simpler than the most complex forms that exist today (Butterfield, 2007). Nervous systems arose early in the animal kingdom and likely had roles in movement and mechanical responses to stimuli. It is much easier to imagine how a simple form of awareness like a single quale could have arisen in early animals if we accept the idea of quantized awareness.

Although V1 represents one highly structured way of organizing qualia, we should not expect that qualia would be arranged in the same way in other animals. Indeed, the striate cortex, as we have come to understand it, is found in only a subset of mammals. Thus the question of the presence or absence of qualia in any specific organism can be separated from the presence or absence of structureslike V1.

We have remained tethered to vision in our discussion of awareness because the visual system is the most thoroughly studied sensory system. If qualia exist for vision, however, they likely exist for other sensory modalities. For instance, the gustatory pathway gives us five distinct types of tastes that could easily correspond to individual qualia. These five are the common sweet, bitter, sour, salty, and umami. The primary gustatory cortex is composed of sections of cortex called the frontal operculum on the inferior frontal gyrus of the frontal lobe and the anterior insula of the insular lobe (Bushnell et al., 2007). Although these regions are not as well understood as V1 they may well contain neural circuits that produce qualia.

Similarly the somatosensory system responds to stimuli from various types of receptors: mechanoreceptors, chemoreceptors, thermoreceptors, and nociceptors. One can imagine either a one to one correspondence with qualia or perhaps a limited number of variant qualia for each. Although it may seem overly simplistic, a natural consequence of the model proposed here is that the complex, rich, and distinct experiences that we humans share arise through varying the number and combinations of a limited set of qualia types. This leads us to a starting point for the creation of a Table of Qualia (**Table 1**).

**Table 1:** Table of sensory qualia.

	Sensory modality				
Qualia type	Visual	Gustatory	Tactile	Auditory	Olfactory
1	Color-1 (red?)	Sour	Mechano	Various evolutionarily	Various evolutionarily
2	Color-2 (blue?)	Salty	Chemo	determined frequency qualia	determined odorant qualia
3	Color-3 (green?)	Umami	Thermo		
4	Motion (various)	Bitter	Pain		
5	Depth	Sweet			
6	Grating orientation (various)				

The descriptions given in this paper are by necessity a first approximation, a starting point. With time and experimentation, the model described in this communication will be reshaped and modified. The true value of the qualia model proposed here is not that it has the final word in how visual awareness comes into being but that it fundamentally changes the questions that can be asked and the approach taken to study visual and other forms of sensory awareness.

## Conflict of Interest Statement

The authors declare that the research was conducted in the absence of any commercial or financial relationships that could be construed as a potential conflict of interest.

## Footnotes

1 The term quale (pl. qualia) is associated with internal perceptions of outer phenomena. For example, the perception of the color blue in your visual field would be considered a quale. Although I maintain this general definition for qualia in this article, I am redefining the scope of a quale to be a quantum of awareness. Included in this redefinition is the assumption that the contribution of a quale is increasingly small compared to our overall visual experience, and that a patch of blue in our field of view would require the integration of many qualia for this percept to be created.

2 The estimate for the number of neurons participating in a circuit that creates a quale is based on estimations of the volume of an ocular dominance column. The cerebral cortex is about 2 mm thick (Hubel etal., 1978), and the average dimensions for ODC is 1 mm (Cheng etal., 2001) by 0.1 mm. The latter dimension is given due to the significant scattering of receptive fields in the movement of electrodes by as little as 0.1 mm. I am assuming neural cells with a cell body diameter of 20 $μ$m (pyramidal cell) and, to simplify, assuming the cells are shaped like cubes.

3 It is clear that many of the philosophical proposals that were previously put forth suffered from a dearth of knowledge of the neuroanatomy and neurophysiology available today about the visual cortices of primates. For instance, the further back one goes in time, one can see that philosophers struggled more often with the question of whether our human awareness was connected to the physiology of the brain or perhaps existed in a state apart from the brain. It has become obvious, that the ideas of Patricia Churchland and others are closer to the true state of the matter in that it appears the functioning of the brain results in awareness. More correctly, it is the activity of certain neural circuits that is visual awareness. The physiological activity and aware state are one and the same – two different aspects of the same process.

4 The qualia model offers a simple means by which the visual cortex can control hue, saturation, and luminance. Hue can be adjusted by changing the relative ratios of red, green, and blue qualia in a percept. Adding triplets of red, blue, and green qualia or removing them as triplets controls illumination. Finally, adding or decreasing the number of red, blue, and green qualia in a percept while maintaining the relative ratios of each governs saturation.
